# Deciphering *SCN2A*: A comprehensive review of rodent models of *Scn2a* dysfunction

**DOI:** 10.1111/epi.18615

**Published:** 2025-08-30

**Authors:** Katelin E. J. Scott, Maria Fernanda Hermosillo Arrieta, Aislinn J. Williams

**Affiliations:** ^1^ Interdisciplinary Neuroscience Graduate Program University of Iowa Iowa City Iowa USA; ^2^ Iowa Neuroscience Institute University of Iowa Iowa City Iowa USA; ^3^ Department of Psychiatry University of Iowa Iowa City Iowa USA; ^4^ Iowa: Discovering Research Experiences and Mentorship Postbaccalaureate Program University of Iowa Iowa City Iowa USA

**Keywords:** autism, epilepsy, Na_v_1.2, therapeutics

## Abstract

*SCN2A* encodes for the alpha subunit of the voltage‐gated sodium channel Na_V_1.2, which is involved in action potential initiation and backpropagation in excitatory neurons. Currently, it is one of the highest monogenetic risk factors for both epilepsy and autism spectrum disorder. However, *SCN2A*‐related disorders manifest in a broad clinical neuropsychiatric spectrum, including distinct neurological and psychiatric disorders. This clinical heterogeneity presents challenges for mechanistic understanding and treatment development. *SCN2A* mutations are generally classified as either gain‐of‐function (GOF) or loss‐of‐function (LOF); however, many mutations do not perfectly align to this binary framework. *SCN2A* dysfunction alters neuronal excitability, channel kinetics, and synaptic transmission in various ways, resulting in multiple electrophysiological effects and both seizure and behavioral phenotypes that are influenced by developmental stage, brain region, genetic background, and sex. Although early lethality in GOF models limits behavioral characterization, LOF models broadly show patterns of learning impairments, altered sociability, and disrupted sensory processing. Still, behavioral and seizure phenotypes are often inconsistent even across models with similar or identical variants, suggesting that genetic modifiers, such as potassium channels, play a role in shaping disease outcomes. Overall, these findings suggest that *SCN2A*‐related disorders involve complex gene–gene and gene–environment interactions, rather than only channel biophysics. Current therapeutic strategies include Clustered Regularly Interspaced Short Palindromic Repeat‐mediated transcriptional activation (CRISPRa), antisense oligonucleotides, and deep brain stimulation; however, they are limited due to variant specificity or age of intervention. This review highlights areas of convergence and conflict across models, emphasizing knowledge gaps, such as the limited availability of data on early development. Ultimately, it emphasizes the importance of investigating models across different developmental stages, using diverse genetic background strains, among other approaches, to encourage therapeutic innovation and enhance care for patients. We hope this work contributes to the emerging unifying framework that looks beyond the GOF and LOF binary in *SCN2A*‐related disorders.

**Key points**

- Rodent models of *SCN2A*‐related disorders reveal that Na_v_1.2 channel dysfunction cannot be fully explained by a simple gain‐ or loss‐of‐function categorization.
- Similar to human *SCN2A* pathology, rodent models of *SCN2A*‐related disorders have heterogeneous phenotypes that warrant closer investigation.
- Rodent models of *SCN2A*‐related disorders have identified promising therapeutic targets and strategies, which have not been fully explored in humans.

## 1 BACKGROUND

*SCN2A‐*related disorders (SRDs) are highly heterogeneous, manifesting in a variety of unique diagnoses, including self‐limited familial and non‐familial infantile epilepsy (SeLFNIE) (previously benign familial infantile seizures),^1^, ^2^ epileptic encephalopathies (EEs),^2^, ^3^, ^4^, ^5^ infantile spasms,^6^ ataxia,^7^ autism spectrum disorder (ASD),^8^, ^9^, ^10^ intellectual disability (ID),^11^, ^12^ and schizophrenia.^11^, ^13^, ^14^ *SCN2A* encodes the alpha subunit of the voltage‐gated sodium channel Na_v_1.2, which is involved in action potential initiation and backpropagation in glutamatergic neurons.^15^ Pathogenic variants of *SCN2A* are typically classified as either gain of function (GOF), which generally implies increased opening of the channel or increased sodium entering through the channel, or loss of function (LOF), where less sodium enters the cell than normal because there is reduced channel expression, or the channel is less functional. Traditionally, GOF variants lead to increased channel activity, whereas LOF lead to decreased activity; however, the channel kinetics of pathogenic *SCN2A* variants alone do not fully predict seizure prognosis and disease severity in SRDs.^1^, ^16^, ^17^, ^18^, ^19^, ^20^, ^21^ There are also some *SCN2A* variants that display characteristics of both GOF and LOF at the same time.^22^, ^23^ These classifications are based on the functional effect a variant has on the Na_v_1.2 channel itself,^24^ often in heterologous expression systems. Variants classified in this manner tend to associate with certain SRD phenotypes (e.g., GOF is often associated with early epilepsy and LOF is associated with neurodevelopmental disorders and autism), but channel kinetics may poorly predict how SRDs manifest in patients.

To investigate this conundrum in SRDs, researchers have utilized non‐human mammalian models to study Na_v_1.2 channel kinetics and associated disorders, which has yielded over 30 rodent models of *Scn2a* dysfunction (Table S1). These efforts have revealed that pathogenic presentation can be heavily influenced by factors beyond channel function, such as background genetics and environment. This review aims to provide a comprehensive evaluation of the current state of non‐human mammalian research on *SCN2A*, the efforts of which have coincided with and complemented human clinical work. A full synthesis of clinical translation goes beyond the scope of this review; however, for an excellent clinical update that includes perspectives from affected families, please see Abbot et al. 2024.^24^ For review of non‐mammalian models of SRDs, please see Hedrich et al., 2019^25^ and Kruth et al., 2020.^26^ Because *SCN2A* variant classification does not always align perfectly with SRD patient phenotypes, this review synthesizes the existing literature beyond the lens of GOF and LOF distinction by first reviewing our current understanding of the cellular functions of Na_v_1.2 to generate a foundation with which to contextualize the non‐human mammalian models of SRDs presented later.

## 2 CELLULAR FUNCTIONS OF NA_V_1.2

Mouse models of SRDs have been instrumental in elucidating the potential roles of *SCN2A* and Na_v_1.2 across brain regions, cell types, and developmental stages. Foundational findings from rodent models of SRDs indicate that Na_v_1.2 is necessary during early development, as homozygous loss of Na_v_1.2 is perinatally lethal.^27^ Na_v_1.2 is largely expressed in glutamatergic excitatory neurons,^15^, ^24^ and heterozygosity of Na_v_1.2 is sufficient to impair synapses and alter glutamatergic neuronal excitability.^28^ Of note, much of our foundational and current knowledge concerning the cellular roles and mechanisms of Na_v_1.2 has been generated from studying neocortical pyramidal cells. Therefore, the data discussed in the following section are derived from that neuron type unless otherwise stated.

### 2.1 How Na_v_1.2 regulates neuron activity at different developmental stages

**FIGURE 1:**
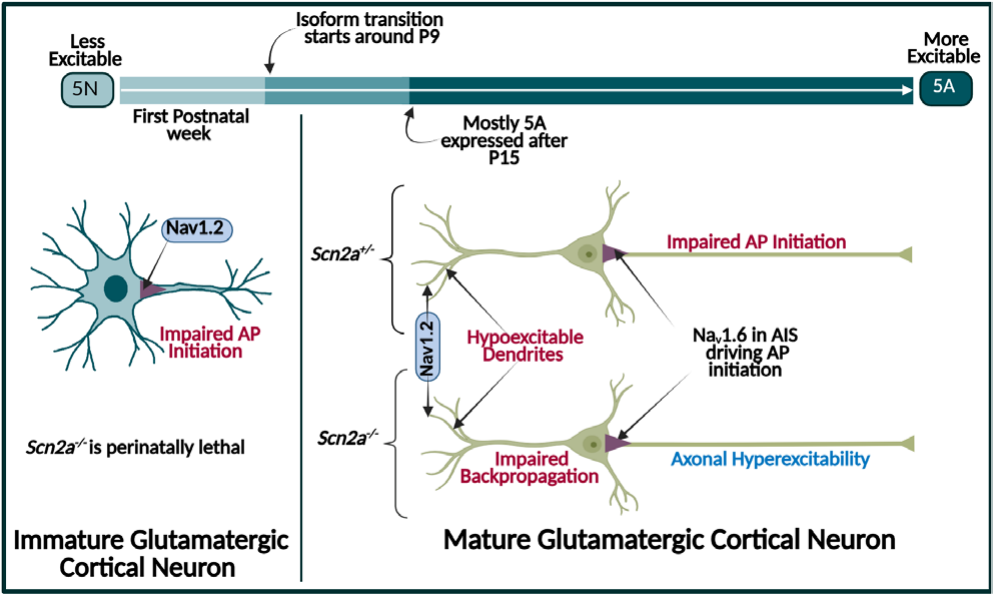
Na_v_1.2 dysfunction impacts glutamatergic cortical neuron activity differently depending on developmental stage. (AP= Acttion Potential, AIS = Axon intial segment)  Created in BioRender. Williams lab, A. (2025) https://BioRender.com/aq9xdz6.

The localization and expression of Na_v_1.2 changes throughout development (Figure 1). *SCN2A* has two major isoforms, neonatal (5N) and adult (5A), which differ by one nucleotide, causing 5A to be more excitable than 5N.^29^, ^30^ The transition between isoforms occurs early in rodents (around postnatal day [P]9^29^), and this neonatal‐to‐adult isoform transition might be one way in which Na_v_1.2 influences the excitability of neurons in developmentally specific ways.^28^, ^30^, ^31^

Na_v_1.2 localization is also developmentally regulated. Na_v_1.2 is located predominantly in the axon initial segment (AIS), at least in myelinated neocortical pyramidal neurons, during the first postnatal week in mice. After the first postnatal week, Na_v_1.6 replaces Na_v_1.2 in the AIS and Na_v_1.2 localizes to dendrites.^24^, ^32^ Immature glutamatergic cortical neurons from *Scn2a* ^*+/−*^ mice have impaired action potential generation.^28^, ^31^ This phenotype is recapitulated when *Scn2a* haploinsufficiency is induced in *Scn2a* ^*+/fl*^ mice via injection of CaMKIIα‐Cre around P50, and these more mature neurons also have decreased dendritic excitability. Complete deletion of Na_v_1.2 in mature glutamatergic cortical neurons causes paradoxical hyperexcitability while still impairing dendritic backpropagation and causing dendritic hypoexcitability. This difference in phenotypes between immature and mature cells, and partial vs complete loss, highlights how Na_v_1.2 has different roles in neuronal excitability in animals during early development vs adulthood.

Deleting *Scn2a* in either forebrain excitatory neurons (*Emx1*‐Cre) or inhibitory neurons globally (*Vgat*‐Cre) in *Scn2a* floxed mice (*Scn2a* ^*fl/fl*^ *or Scn2a* ^*+/fl*^) reveals additional age‐dependent outcomes.^33^, ^34^ Homozygous loss of Na_v_1.2 in either cell type was sufficient to cause perinatal lethality,^34^, ^35^ demonstrating that Na_v_1.2 is required in inhibitory as well as excitatory neurons during early postnatal development. However, in adult mice, Na_v_1.2 expression was reduced in the neocortex and hippocampus of *Scn2a* ^*+/fl*^ */Emx1‐*Cre mice but not *Scn2a* ^*+/fl*^ */Vgat‐*Cre mice,^34^, ^35^ suggesting that at least in the cortex and hippocampus, Na_v_1.2 is mainly expressed in excitatory neurons. The activity of cortical inhibitory neurons was largely unaltered by reduction of Na_v_1.2.^34^, ^35^ Yet, about a third of *Scn2a* ^*+/fl*^ */Vgat‐*Cre mice died between P18 and P25 of unknown causes, indicating that Na_v_1.2 may be necessary in some inhibitory cells during later juvenile development. It is also possible that inhibitory cells outside of the cortex, which were affected but not investigated in this model, may be more greatly affected by loss of Na_v_1.2.^34^ *Scn2a* is also known to be expressed in a variety of non‐cortical cell types, some of which are reviewed below.

### 2.2 Na_v_1.2‐mediated regulation of somatodendritic excitability

As mentioned above, Na_v_1.2 is localized to dendrites in mature neurons, and loss of Na_v_1.2 causes reduced dendritic backpropagation. Another potential mechanism by which Na_v_1.2 regulates somatodendritic excitability is through its close and coordinated interactions with voltage‐gated potassium (K_v_) channels. When open, Na_v_1.2 channels provide a depolarizing current, which shifts the membrane potential and readies K_v_ channels to open and repolarize the neuron.^36^, ^37^ Reduced dendritic backpropagation caused by a lack of dendritic Na_v_1.2 would result in fewer voltage‐gated potassium channels opening during repolarization.^28^, ^31^ This would reduce the refractory period and allow for the generation of more action potentials while simultaneously impairing repolarization.

This interaction with K_v_ channels is likely not the only mechanism by which alterations in Na_v_1.2 can lead to changes in neuronal excitability. For instance, the *SCN2A*‐p.K1422E variant (*Scn2a* ^*+/K1422E*^), which is located in the channel pore, disrupts the ion selectivity filter of Na_v_1.2 and displays characteristics of both LOF and GOF.^22^ This variant causes the channel to become non‐selective for cations, which reduces sodium conductance, alters repolarization, and impairs neuronal firing.^22^ Loss of cation selectivity appears to be a large contributing factor, leading to slower action potential initiation in a mouse model of this variant.^22^ Other work has shown that when antisense oligonucleotides (ASOs) are used to reduce *Scn2a* expression in the cortex, layer 5 (L5) pyramidal neurons display reduced Ca^2+^ transient currents.^38^ Reduction of these currents results in decreased spontaneous cortical somatosensory neuronal firing and pairwise co‐activation, similar to reduced overall neuronal excitability in immature cortical neurons from *Scn2a* ^*+/−*^ mice.^28^, ^38^ Thus, when Na_v_1.2 does not work properly, it has broad impacts on neuronal physiology.

### 2.3 Post‐translational modifications

Another potential mechanism by which changes in *SCN2A* can alter neuronal excitability is via regulation of channel SUMOylation.^39^ The *Scn2a* ^*K38Q*^ mouse carries a point mutation at amino acid 38, which switches Lys for Gln, thereby removing the only known SUMO‐conjugation site in Na_v_1.2.^39^ SUMOylation at this site, which occurs during periods of hypoxia, increases sodium current through Na_v_1.2.^40^ The *Scn2a* ^*K38Q*^ mouse revealed that SUMOylation of Na_v_1.2 channels prolongs the decay time constant of excitatory postsynaptic potentials (EPSPs) greater than 10 mV in amplitude and reduces the speed of forward and backpropagating action potentials of cortical pyramidal cells.^39^ Pathogenic *SCN2A* mutations that disrupt SUMOylation of the Na_v_1.2 channel may therefore cause reduced action potential propagation speed, frequency, and/or amplitude. This also raises the possibility that mutations that allow for additional SUMOylation sites or alter other post‐translational modifications could change these electrophysiological features. These findings indicate that changes in SUMOylation and likely other post‐translational modifications (Na_v_1.2 can have many; reviewed in Onwuli & Beltran‐Alvarez, 2016^41^) can impact channel kinetics, and thus can alter the presentation of signs and/or symptoms of SRDs.

### 2.4 Beyond the cortex

Although Na_v_1.2 has been studied mostly in the cortex, it is widely expressed throughout the brain. For example, Na_v_1.2 has been identified in cerebellar granule cells (CGNs), which are unmyelinated glutamatergic excitatory neurons, the function of which can be assayed using the vestibular ocular reflex (VOR).^42^ VOR stabilizes vision when the head moves.^42^ In *Scn2a* ^*+/−*^ mice, VOR gain, or the ratio of eye movements to head movement, is significantly greater, suggesting an over‐responsive reflex.^42^ Removing just one copy of *Scn2a* only in CGNs was sufficient to enhance VOR gain, indicating that Na_v_1.2 in CGNs is involved, if not necessary, for proper expression of this reflex.^42^ VOR changes in *Scn2a* mouse models parallel the changes seen in some humans with SRDs.^42^, ^43^ Altered VOR gain is likely due to changes in the synapses between CGNs and Purkinje cells, but this has not been confirmed.

**FIGURE 2:**
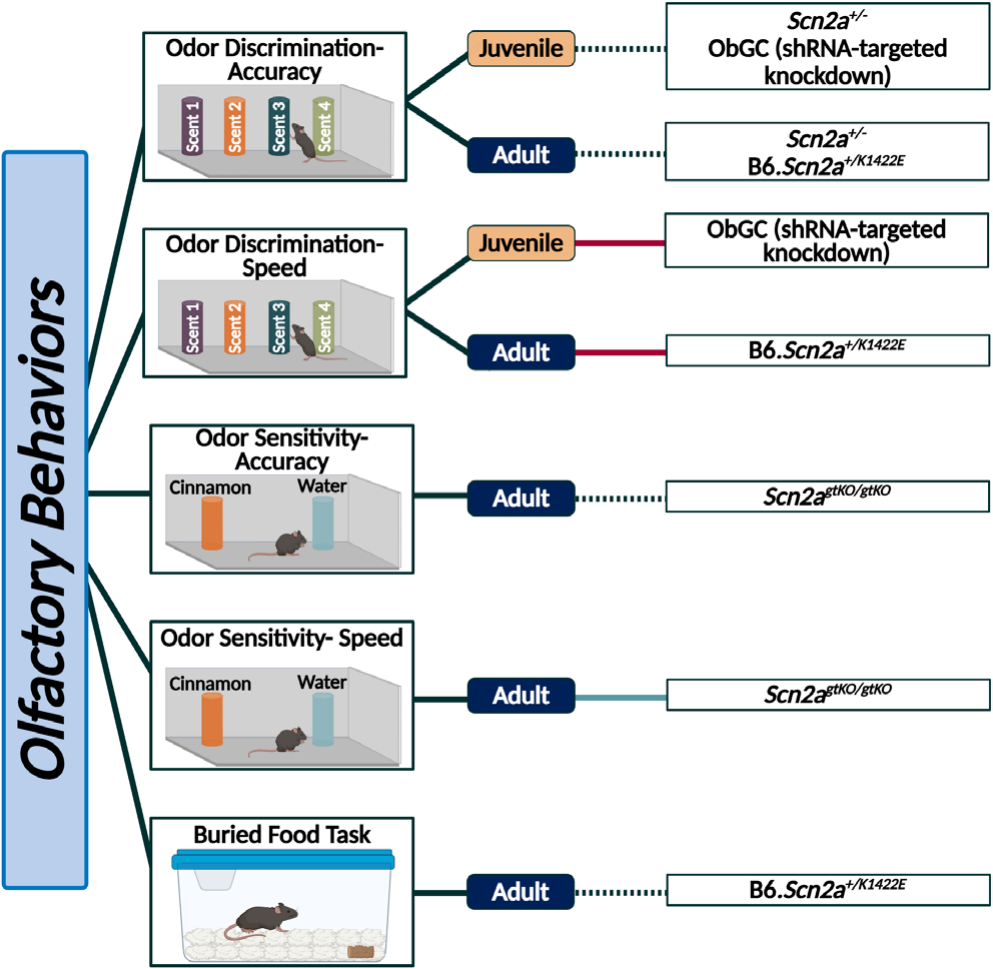
Summarized findings of olfaction‐related behaviors in *Scn2a* mouse models. Behaviors are separated by age at time of assessment. Blue lines indicate an increase in the behaviors observed in that assay, red lines indicate a decrease in the behaviors observed in that assay, and black small‐dotted lines indicate no change. Large‐dashed lines are utilized to indicate trends and colored red or blue to indicate the direction of the trend. Created in BioRender. Williams lab, A. (2025) https://BioRender.com/pglh067.

Na_v_1.2 is also important in inhibitory neurons outside of the cortex. Knockdown of Na_v_1.2 in olfactory bulb granule cells (ObGCs), which are unmyelinated γ‐aminobutyric acid (GABA)ergic inhibitory interneurons, reduces the speed of odor discrimination but does not affect accuracy (Figure 2, Table S2).^44^ This phenotype is thought to be secondary to inhibition of synaptically connected mitral cells, diminished GABA release, and reduced ObGC spiking.^44^ *Scn2a* ^*gtKO/gtKO*^ mice (also called “gene trap” mice), which express ≤25% of normal Na_v_1.2, also have slower odor discrimination, whereas *Scn2a* ^*+/−*^ mice do not, suggesting that between 25% and 50% of normal Na_v_1.2 levels are required for rapid odor discrimination but not for accuracy (Figure 2).^45^, ^46^ Furthermore, another inhibitory cell type, striatal medium spiny neurons, display increased neuronal excitability in adult *Scn2a* ^*gtKO/gtKO*^ mice, similar to the paradoxical hyperexcitability of cortical pyramidal neurons lacking Na_v_1.2.^31^, ^47^ These data further indicate that Na_v_1.2 can have an integral role in inhibitory cells.

Investigation of Na_v_1.2 in the hippocampus, a subcortical structure, has identified roles for the channel that may be relevant for human phenotypes. Multiple *Scn2a* models, typically GOF, present with greater hippocampal activity than wild‐type mice, which often drives their seizure presentation.^48^, ^49^, ^50^ The F1.*Scn2a* ^*Q54*^ mouse model (described later) is prone to hilar neuronal loss, indicating that the hippocampus may be sensitive to the effects of some *Scn2a* variants, especially those that generate seizures.^51^ Conversely, hippocampal cultures from *Scn2a* ^*+/−*^ mice have reduced sodium currents compared to wild‐type cells.^27^ In addition, hippocampal long‐term potentiation was suppressed in *Scn2a* ^*+/−*^(Δ4‐6) mice without affecting long‐term depression, and *Scn2a* ^*gtKO/gtKO*^ mice have reduced synaptic transmission and lower spine density in hippocampal neurons, which may be linked to their impaired learning and memory.^52^, ^53^ All these data together suggest that Na_v_1.2 is playing an integral role in this structure.

Little is known about Na_v_1.2 expression and function in non‐neuronal cell types. One study suggests that Na_v_1.2 is necessary for spiking activity and maturation of a subset of oligodendroglia in the brainstem and cerebellum.^54^ Early postnatal deletion of Na_v_1.2 in *Scn2a* ^*fl/f*^ */Actin‐*Cre^ERT^ mice effectively removed spiking oligodendroglia without impacting differentiation and maturation of other oligodendroglia.^54^ *Scn2a* loss has also been associated with microglial activation and excessive pruning of neuronal processes.^53^ The potential role and expression of Na_v_1.2 in other non‐neuronal cell types should continue to be investigated in models of SRDs. Having a better understanding of Na_v_1.2 function in diverse cell types will contribute to our understanding of why and how pathogenic *SCN2A* variants lead to such heterogeneous phenotypes.

## 3 SEIZURES AND *SCN2A* MOUSE MODELS

Seizures occur commonly in SRDs, and mouse models of *Scn2a* have emulated some seizure phenotypes.^10^, ^22^, ^34^, ^50^, ^51^, ^55^, ^56^ Most mouse models with seizures display tonic–clonic and other convulsive‐type seizures,^50^, ^51^, ^56^, ^57^ although a few models have absence‐like seizures.^33^, ^34^ Evaluation of seizures across *Scn2a* mouse models has revealed variable degrees of seizure susceptibility and severity depending on both the specific *Scn2a* mutation and the genetic background or strain of the mouse model.^48^, ^49^, ^57^

### 3.1 Strain effects

Strain‐dependent effects of seizure phenotype are best illustrated by data from *Scn2a* ^*Q54*^ and *Scn2a* ^*+/K1422E*^ mice.^48^, ^49^, ^57^ *Scn2a* ^*Q54*^ mice display spontaneous seizures that originate in the hippocampus and increase in number and duration with age, eventually leading to premature death.^51^, ^58^ Originally, the *Scn2a* ^*Q54*^ mouse was engineered on a mixed C57BL/6J x SJL/J (F1.*Scn2a* ^*Q54*^) background but was later backcrossed to a congenic C57BL/6J background (B6.*Scn2a* ^*Q54*^), which attenuated the seizure phenotype.^48^, ^49^, ^59^ B6.*Scn2a* ^*Q54*^ mice display increased hippocampal activity compared to wild‐type mice, but this phenotype is even more pronounced in F1.*Scn2a* ^*Q54*^ mice, leading to a more severe seizure phenotype.^59^ *Scn2a* ^*+/K1422E*^ mice also show strain‐specific seizure susceptibility. *Scn2a* ^*+/K1422E*^ mice, however, have infrequent and spontaneous seizures localized to the posterior cortex instead of the hippocampus.^22^ *Scn2a* ^*+/K1422E*^ mice were created on the C57BL/6J background (B6.*Scn2a* ^*+/K1422E*^) but were then crossed with DBA/2 J mice to generate a hybrid background (F1D1.*Scn2a* ^*+/K1422E*^).^57^ B6.*Scn2a* ^*+/K1422E*^ mice are more susceptible to kainic acid (KA)–induced seizures with evident sex differences in seizure phenotype, whereas F1D1.*Scn2a* ^*+/K1422E*^ mice have comparably less severe KA seizures and no sex differences.^57^ Of interest, both background strains have low penetrance of spontaneous convulsive seizures.^57^ These data suggest that background strain selection is an important consideration for SRD model development.

### 3.2 Environmental and developmental effects

*SCN2A* GOF mutations are associated with neonatal seizure onset in humans.^10^, ^16^, ^17^ *Scn2a* ^*+/A263V*^ and *Scn2a* ^*+/R1883Q*^ are GOF mouse models that carry variants first identified in human patients and have been used to evaluate seizure phenotypes.^50^, ^56^ The *Scn2a* ^*+/R1883Q*^ mouse was generated by inserting the point mutation p.R1883Q into exon 26 of the *Scn2a* gene.^56^ These mice display phenotypes consistent with developmental epileptic encephalopathy and die by P30 due to seizures.^56^ The *Scn2a* ^*+/A263V*^ model, like *Scn2a* ^*Q54*^, displays increased activity in hippocampal CA1 pyramidal neurons; however, *Scn2a* ^*+/A263V*^ mice do not have impaired survival.^50^ Of note, the *Scn2a* ^*+/A263V*^ model is on a congenic C57BL/6J background, the same genetic background that attenuated seizure severity in *Scn2a* ^*Q54*^ mice.^48^, ^50^

Approximately 30% of patients with *SCN2A* LOF mutations also develop seizures, generally at later ages than GOF patients.^10^, ^17^, ^24^ This parallels clinical data suggesting that the electrophysical properties of *SCN2A* variants in heterologous expression systems are poor predictors of disease severity, as channel kinetics are unable to predict seizure prognosis.^1^, ^16^, ^17^, ^18^, ^19^, ^20^, ^21^ Some Na_v_1.2 LOF models, such as *Scn2a* ^*+/−*^ and *Scn2a* ^+/R102X^ (which also models a human variant), have spontaneous electrographic absence‐like seizures. Loss of Na_v_1.2 in forebrain L5 excitatory neurons was sufficient to cause these seizures.^33^, ^34^ *Scn2a* ^+/R102X^ mice also have infrequent spontaneous convulsive seizures,^34^ whereas *Scn2a* ^*+/−*^ mice have been documented to have convulsive seizures only after water restriction.^55^ This suggests that *Scn2a* seizure semiology can be influenced by both experience and the channel variant present. Other LOF models, like *Scn2a* ^*+/−*^(Δ4‐6), show changes in excitatory neuronal activity but no spontaneous seizure activity.^52^ Changes in neuronal excitability caused by severe Na_v_1.2 reduction, such as paradoxical hyperexcitability, are hypothesized to contribute to seizures experienced by individuals with *SCN2A* LOF mutations.

*Scn2a* isoforms also influence seizure phenotype. As mentioned, the two major isoforms of *SCN2A*, 5N and 5A, have different electrophysical properties.^29^, ^30^ When neonatal mice express 5A (the *Na*_*v*_ *1.2* ^*Adult*^ model), the mice are hyperactive and have seizures.^30^ This seizure phenotype may be similar to some human *SCN2A* patients with GOF variants who experience seizures early in life that remit with age.^30^, ^60^, ^61^ These GOF variants may cause 5N (less excitable isoform) to behave more like 5A (more excitable isoform), and seizures attenuate later in life when the Na_v_1.2 isoform activity better matches the developmental stage.

### 3.3 Genetic modifiers

The C57BL/6J and C57BL/6N strains have distinct modifier alleles that influence the severity of seizure phenotypes and survival. C57BL/6J mice are generally more resistant to chemoconvulsant seizures.^62^, ^63^ Although seizure activity in *Scn2a* GOF models (e.g., *Scn2a* ^*Q54*^, *Scn2a* ^*+/A263V*^, *Scn2a* ^*+/R1883Q*^) is of similar hippocampal origins, mortality is heavily influenced by the background strain of the model.

**FIGURE 3:**
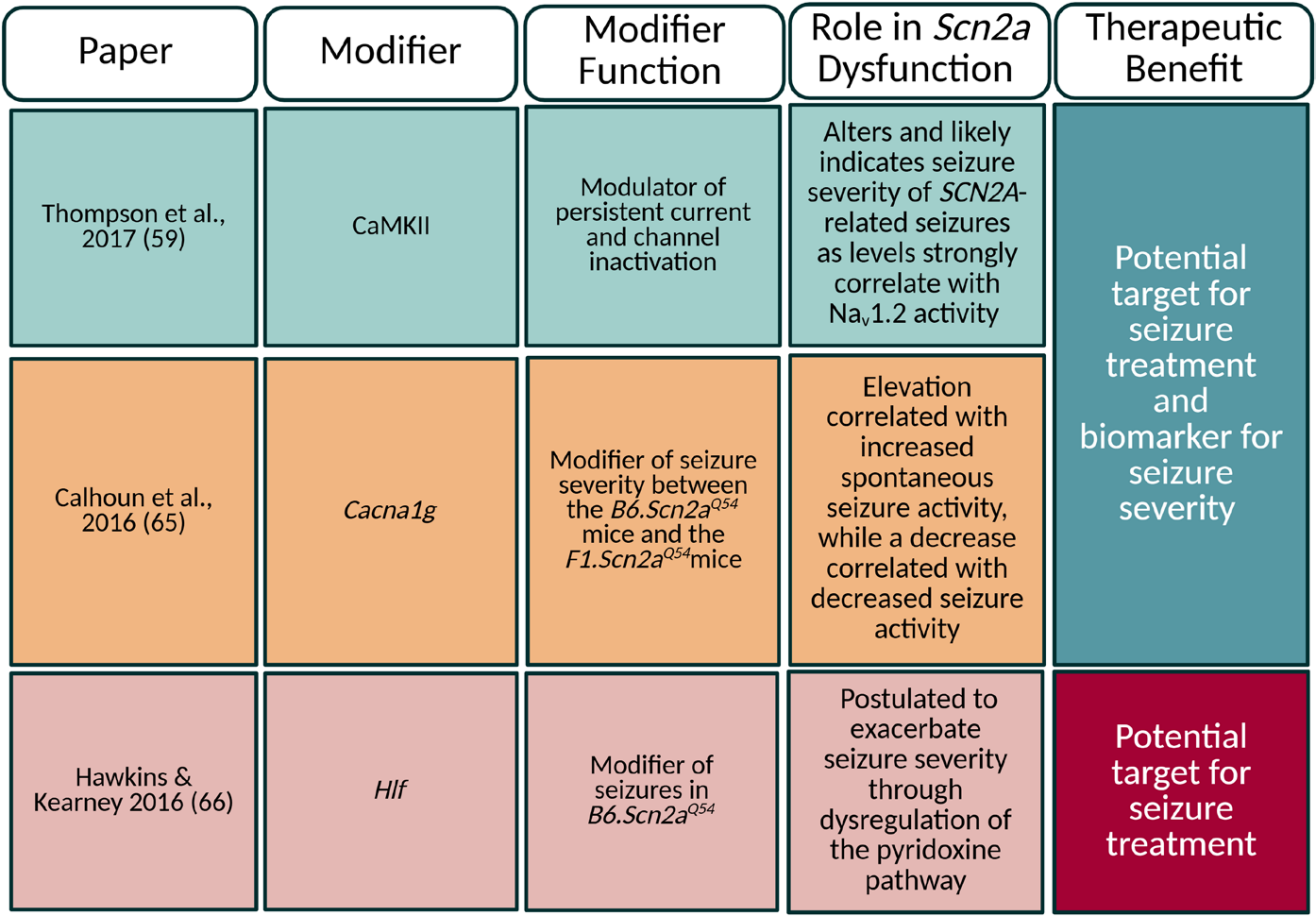
Table of additional modifiers identified in *Scn2a* ^*Q54*^ mice. Created in BioRender. Williams lab, A. (2025) https://BioRender.com/l21j948.

Some efforts have been made to identify what specifically drives the differences in seizure phenotypes in these models. The SJL background strain, used for some GOF animals, carries a native mutation that suppresses the expression of Dickkopf‐1 (*Dkk1*), a Wnt signaling inhibitor.^48^ However, reducing levels of *Dkk1* in B6.*Scn2a* ^*Q54*^ mice by crossing them with *Dkk1* ^*+/−*^ mice did not increase seizure susceptibility or incidence.^48^ Furthermore, genetic screening identified loci on chromosomes 11 and 19, Modifier of epilepsy 1 (Moe1), and Modifier of epilepsy 2 (Moe2), respectively, which accounted for 80% of the seizure phenotype differences between B6.*Scn2a* ^*Q5*^ and F1.*Scn2a* ^*Q54*^ mice; both are downstream of *Dkk1*.^48^, ^64^ Careful chromosome mapping identified 24 other potential genetic modifiers within the Moe2 locus.^49^ Sequencing of the candidate genes uncovered non‐synonymous coding sequence polymorphisms in the potassium channel gene *Kcnv2* (the voltage‐gated potassium channel, K_v_8.2) and the transcription factor *Smarca2*.^49^ Ultimately, *Kcnv2* was determined to be the strongest functional candidate within the Moe2 locus for mediating seizure phenotype differences between F1.*Scn2a* ^*Q54*^ and B6.*Scn2a* ^*Q54*^ mice.^49^ Other potential genetic modifiers are listed in Figure 3.^59^, ^65^, ^66^ Although these genetic modifiers were identified in *Scn2a* ^*Q54*^ mice, other modifiers likely occur in other *Scn2a* models and merit further investigation. Identifying genetic modifiers might provide insight into why individuals with identical mutations present with clinically distinct symptomatology and severity.^16^, ^67^

Generating a given *Scn2a* variant on multiple genetic backgrounds may help to elucidate which phenotypes are caused by the variant and which are influenced by genetic or epigenetic modifiers. For instance, *Scn2a* ^*+/K1422E*^ were found to have more resistance to seizure generalization and greater death following convulsive seizures compared to wild‐type, regardless of background strain, whereas sex differences in seizure phenotypes were background strain‐dependent.^57^ Current data further indicate that the C57BL/6J background may attenuate seizures for some *Scn2a* variants^48^, ^49^, ^50^; clearly, this could be a hindrance if the goal is to study seizures. However, seizure attenuation could also be viewed as a benefit, since this may enable investigations of non‐seizure or age‐dependent phenotypes in models that display early mortality on other genetic backgrounds.^48^, ^49^ This becomes very important in clinical conditions like SRDs because there are many medical issues beyond seizures that impair patients' quality of life, such as sleep disturbances and dysautonomia, about which we know very little and for which we have no effective treatments.

### 3.4 *Scn2a* and potassium channels

The voltage‐gated potassium channel K_v_7.2 contributes to seizure phenotypes in *Scn2a* ^*Q54*^ mice.^68^ This potassium channel generates M currents that modulate hippocampal neuron firing patterns and excitability.^68^ Loss of K_v_7.2 function exacerbates seizure severity in *Scn2a* ^*Q54*^ mice, resulting in death around P21.^68^ The intense seizure phenotype of the double‐mutant mice suggests that M currents play an important role in modulating seizure initiation and spreading^69^ and that targeting K_v_7.2 may alleviate *SCN2A*‐related seizures. As mentioned earlier, K_v_8.2 may also exacerbate seizure severity in *Scn2a* ^*Q54*^ mice and is another potential therapeutic target.^59^

Potassium channels also modulate *Scn2a* LOF phenotypes.^70^, ^71^ Mice heterozygous for both Na_v_1.2 and K_V_1.1, *Scn2a* ^*+/−*^ *Kcna1* ^*+/−*^ mice, have improved phenotypes compared to both *Scn2a* ^*+/−*^ and *Kcna1* ^*−/−*^ mice, respectively.^70^, ^71^ Moreover, K_V_1.1 and other potassium channels may contribute to the hyperexcitability of S*cn2a* ^*gtKO/gtKO*^ medium spiny neurons.^47^ Exposure to 4‐trifluoromethyl‐L‐phenylglycine, an agonist for K_v_1.1, and pimaric acid, a global potassium channel agonist, reduce hyperexcitability in S*cn2a* ^*gtKO/gtKO*^ medium spiny neurons.^47^ Transcriptomic data from *Scn2a* ^*gtKO/gtKO*^ brain identified multiple downregulated potassium channels in response to loss of Na_v_1.2, including *Kcne2*, *Kcng4*, *Kcnv1*, *Kcna1* (K_v_1.1), *Kcna2* (K_v_1.2), *Kcnj10*, and *Kcnk1*.^47^ These data together indicate that compensatory and maladaptive changes in potassium channels may contribute to seizure onset in *Scn2a* LOF mice. This suggests that Na_v_1.2 and potassium channels have a dynamic relationship that may be helpful from a therapeutic perspective.^31^, ^47^

## 4 BEHAVIORAL PHENOTYPES ACROSS *SCN2A* MOUSE MODELS

Dysfunction of *SCN2A* in humans leads to a plethora of behavioral signs and symptoms. Rodent models of SRDs capture some of these heterogeneous features. *Scn2a* rodent models have been assessed with many behavioral assays, and they frequently demonstrate overlapping phenotypes, but specific behavioral changes and phenotypes can be variable across investigations and models.^22^, ^33^, ^34^, ^38^, ^42^, ^44^, ^45^, ^46^, ^52^, ^54^, ^55^, ^57^, ^70^, ^71^, ^72^, ^73^, ^74^, ^75^, ^76^, ^77^, ^78^

**FIGURE 4:**
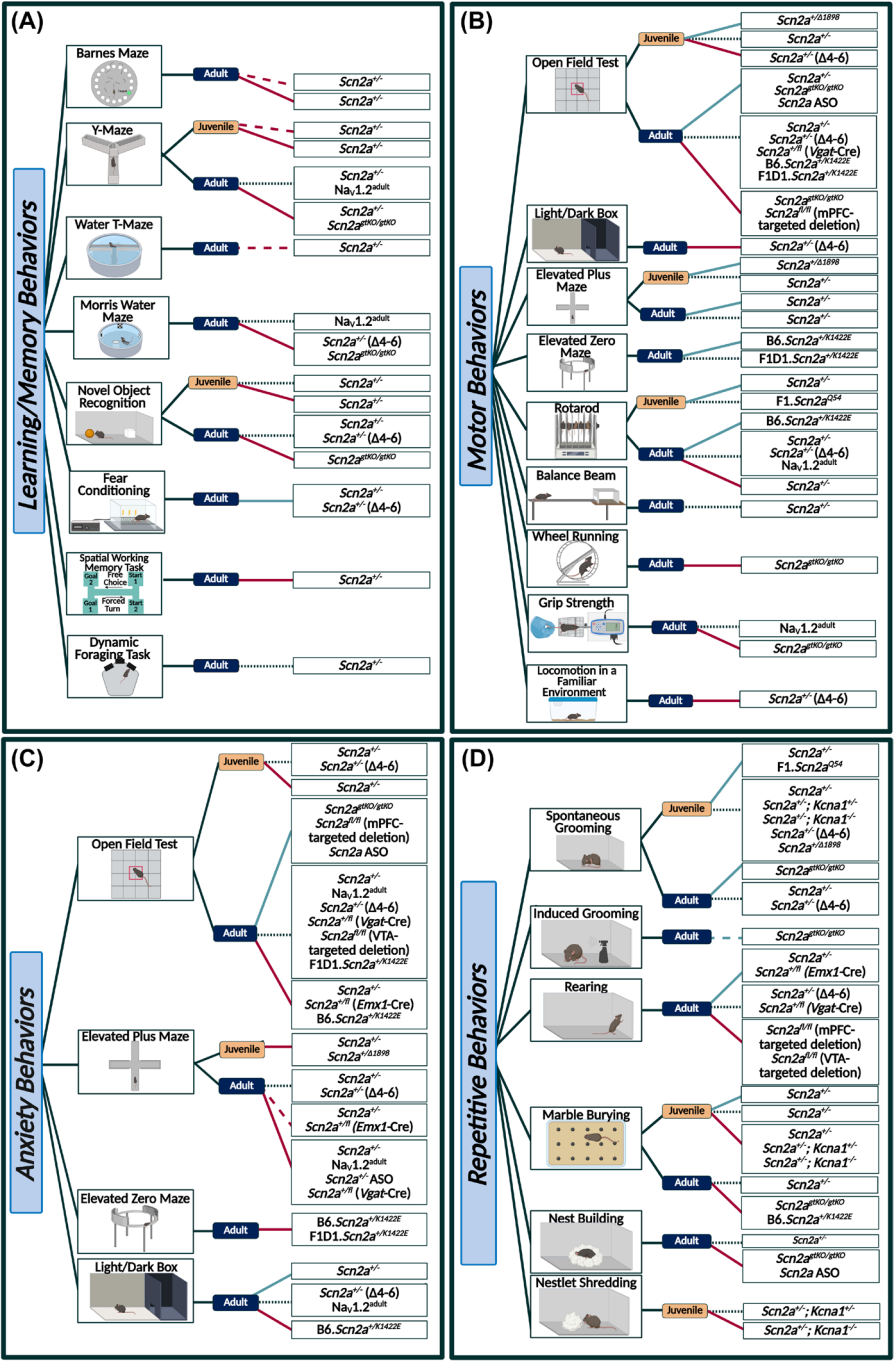
(A) Summary of results from learning and memory related assays in *Scn2a* mouse models. (B) Summary of results from motor behavior related assays in *Scn2a* mouse models. (C) Summary of results from anxiety‐like behavioral assays in *Scn2a* mouse models. (D) Summary of results from assays related to repetitive behaviors in *Scn2a* mouse models. Behaviors in A, B, C and D are broken down by age at time of assessment. Blue lines indicate an increase in the behaviors observed in that assay, red lines indicate a decrease in the behaviors observed in that assay, and black small‐dotted lines indicate no change. Large‐dashed lines are utilized to indicate trends and colored red or blue to indicate the direction of the trend. Created in BioRender. Williams lab, A. (2025) https://BioRender.com/sa0eb87.

Assays related to learning and memory appear to demonstrate general consistency across models, largely showing reduced performance in mice with decreased *Scn2a* expression, both at adult and juvenile ages (although the juvenile data are sparser) (Figure 4A).^28^, ^30^, ^45^, ^46^, ^52^, ^53^, ^55^, ^57^, ^73^, ^75^ Fear conditioning, however, shows increased freezing in two distinct *Scn2a* LOF models.^52^, ^74^ Tests of locomotor function and learning are more mixed, with a slight predominance of results favoring reduced locomotor function in *Scn2a* LOF mice (Figure 4B).^28^, ^30^, ^38^, ^45^, ^46^, ^52^, ^72^, ^74^, ^75^, ^76^, ^77^ Of interest, *Scn2a* ^*+/K1422E*^ mice, where the mutation causes loss of channel filter selectivity, appear to show increased locomotor activity on some assays.^22^, ^57^ Many *Scn2a* mouse models have undergone testing for anxiety or exploratory behavior, as well as restricted and repetitive behavior, but the results for these assays are quite mixed and preclude generalization. Juvenile *Scn2a* LOF mice predominantly show reduced anxiety‐like behaviors,^45^, ^70^, ^72^, ^75^ whereas assays in adult *Scn2a* LOF mice are inconsistent (Figure 4C).^22^, ^28^, ^30^, ^38^, ^45^, ^46^, ^52^, ^57^, ^72^, ^74^, ^75^, ^77^ Repetitive behaviors are also variable between *Scn2a* LOF mice and assay type (Figure 4D).^22^, ^28^, ^38^, ^45^, ^46^, ^52^, ^70^, ^72^, ^74^, ^75^, ^77^

**FIGURE 5:**
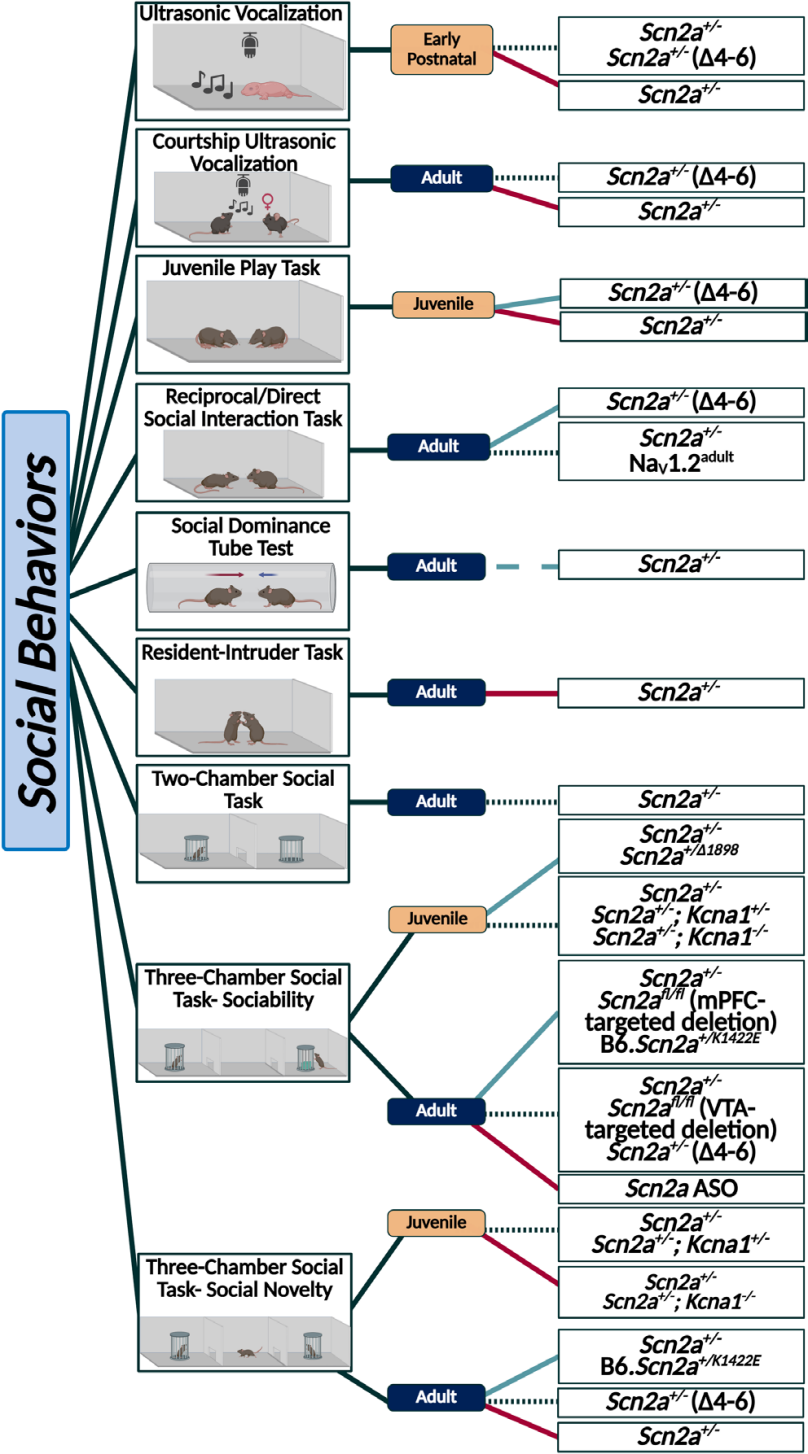
Summary of results related to sociability and social novelty in *Scn2a* mouse models. Behaviors are separated by age at time of assessment. Blue lines indicate an increase in the behaviors observed in that assay, red lines indicate a decrease in the behaviors observed in that assay, and black small‐dotted lines indicate no change. Large‐dashed lines are utilized to indicate trends and colored red or blue to indicate the direction of the trend. *Created in BioRender*. Williams lab, A. (2025) https://BioRender.com/rfd40nf.

Social behaviors are a good example of how behaviors can be dissimilar across studies, even in the same model. For instance, the *Scn2a* ^*+/−*^ mouse, where exon 1 of the *Scn2a* gene is disrupted, generates conflicting results in different social experiments (Figure 5).^27^, ^28^, ^33^, ^34^, ^42^, ^45^, ^55^, ^70^, ^72^, ^73^, ^74^, ^75^, ^76^ Some investigations found increased sociability in the three‐chamber social interaction test,^71^, ^74^ whereas others reported no changes.^70^ In the reciprocal social interaction test and the two‐chamber social task, *Scn2a* ^*+/−*^ mice had decreased sociability, sometimes in a sex‐ and age‐specific manner.^28^, ^45^ *Scn2a* ^*+/Δ1898*^ mice, which carry a 27‐base pair frameshift in the 3′ end of the *Scn2a* gene, also have sex‐specific differences in social behaviors, and *Scn2a* ^*+/−*^(Δ4‐6) mice display age‐dependent changes to sociability and communication.^22^, ^52^, ^72^ Of interest, deleting *Scn2a* from the ventral tegmental area (VTA),^77^ crossing *Scn2a* ^*+/−*^ mice with mice lacking one or both copies of *Kcna1* (*K*_*v*_ *1.1*),^70^ a voltage‐gated potassium channel, and the *Na*_*v*_ *1.2* ^*Adult*^ model, which expresses the adult isoform of *Scn2a* (5A) from birth,^30^ showed no changes in sociability behaviors.^70^, ^71^ It is important to note that although no differences in sociability were identified in *Na*_*v*_ *1.2* ^*Adult*^ mice, they were 16 times more likely to try to escape the interaction chamber.^30^ Finally, deletion of *Scn2a* from the medial prefrontal cortex of *Scn2a* ^*fl/fl*^ mice leads to increased sociability, similar to some evaluations of *Scn2a* ^*+/−*^ mice, suggesting that this brain region might be particularly important for sociability phenotypes of Na_v_1.2 loss.^77^ Conversely, reduced sociability phenotypes are reported in mice that have widespread *Scn2a* knockdown via injection of ASOs.^38^ Together, these findings suggest that social behaviors of SRD mouse models are age‐, sex‐, and assay‐specific.

Other behavioral phenotypes identified in some models, which have not been verified across others, include impaired VOR gain and sleep disruption (Table S2).^42^, ^76^ VOR and sleep changes could both potentially serve as non‐invasive measurements to track disease severity and treatment response in humans, as VOR and sleep appear to be sensitive to at least some pathogenic *SCN2A* variants.^42^, ^76^ Of note, the model used to evaluate sleep, the *Scn2a* ^*gtKO/gtKO*^ mouse, was generated on the C57BL/6N strain.^46^, ^76^ Most models, however, are on the C57BL/6J background.^28^, ^30^, ^33^, ^34^, ^42^, ^45^, ^52^, ^55^, ^70^, ^71^, ^73^, ^74^, ^75^ This situation creates a unique predicament, as the C57BL/6N strain may be more sensitive than other strains to sleep changes, like alterations in core clock genes and disrupted sleep patterns demonstrated by the *Scn2a* ^*gtKO/gtKO*^ mouse.^46^, ^53^, ^76^, ^79^ This situation underscores an urgent need to investigate sleep and other behaviors in *Scn2a* models across multiple genetic backgrounds, since it is unknown which background strain most accurately and reliably recapitulates human SRD pathology.

It is notable that all models that have undergone significant behavioral testing, except *Scn2a* ^*+/K1422E*^ and *Na*_*v*_ *1.2* ^*Adult*^ mice, are some variation of LOF.^22^, ^28^, ^33^, ^34^, ^38^, ^45^, ^70^, ^71^, ^72^, ^73^, ^74^, ^75^, ^80^ Of interest, however, the three most similar LOF models discussed, *Scn2a* ^*+/−*^, *Scn2a* ^*+/−*^ (Δ4‐6), and *Scn2a* ^*+/Δ1898*^, have distinct behavioral phenotypes that differ from one another as exemplified by the discussion of sociability (Figure 5).^28^, ^33^, ^34^, ^45^, ^52^, ^55^, ^70^, ^71^, ^72^, ^73^, ^74^ The *SCN2A* variant carried by the *Scn2a* ^*+/Δ1898*^ model could theoretically occur in humans, whereas the mutations carried by *Scn2a* ^*+/−*^ and *Scn2a* ^*+/−*^ (Δ4‐6) mice do not naturally occur.^27^, ^52^, ^72^ This raises two possibilities: (1) models that carry a plausible or identified human *SCN2A* variant are more representative of human phenotypes, or (2) *Scn2a* mouse models, like human *SCN2A* patients, are heterogeneous in their symptom profile. The data above suggest that the latter is more likely. Each mouse construct, regardless of similarity of mutation or plausibility of occurrence in humans, is likely distinct in presentation from one another, as demonstrated by the complex and oftentimes contrasting behavioral results (Figures 2, 4 and 5; Table S2). This possibility is rather exciting, as it means that *Scn2a* mutations alone are unlikely to be the sole driving factor of the phenotypes observed in mouse models. This parallels human data, which suggest that pathogenic *SCN2A* variants present with distinct and unique symptomatology across patients with identical mutations.^16^, ^67^ The variability in both human and mouse data highlights the importance of continuing to identify classes of genetic modifiers that contribute to the heterogeneous nature of SRDs and can serve as potential therapeutic targets.

Finally, the behaviors of GOF *Scn2a* models have not been discussed in this section. This is because the current GOF *Scn2a* mouse models have severe seizure phenotypes and die prematurely, preventing most traditional behavioral studies.^50^, ^51^, ^56^, ^58^ However, *Scn2a* ^*Q54*^ mice, which express FLAG‐tagged *Scn2a* GAL879‐881QQQ in the conserved S4‐S5 linker region of domain 2 under the neuron‐specific enolase promoter, were observed to have typical motor coordination (Figure 4B).^51^ Atypical grooming behaviors have also been observed in these mice (Figure 4D).^51^, ^58^ With that said, it is currently unknown how heterogeneous behavioral phenotypes would be across other GOF mouse models or if the behaviors would be comparable to LOF models, as behavior beyond seizures has been largely impossible to explore.

## 5 TREATMENT

### 5.1 Genetic rescue

Work is ongoing to develop viable and safe genetic therapies to alleviate SRDs. One effort uses Clustered Regulatory Interspaced Short Palindromic Repeat‐mediated transcriptional activation (CRISPRa), which utilizes deactivated Cas9 (dCas9) fused to a transcriptional coactivator to increase expression of *Scn2a* for LOF variants.^80^ Preclinical work shows that CRISPRa‐based treatment restored typical excitability patterns in *Scn2a* ^*+/−*^ cortical neurons and did not induce seizures or lead to phenotypic abnormalities in mice.^80^ CRISPRa attenuated the hypersensitive VOR gain when administered at P3 and at P30, although only the P3 treatment restored baseline VOR.^42^ Based on these results, CRISPRa therapy may be promising for *SCN2A* LOF variants, with the greatest impact at very young ages, but later administration may still provide some therapeutic benefit.^30^, ^42^ This study did not test intermediate ages between P3 and P30; however, it would be interesting to know whether the neonatal/adult isoform switch of Na_v_1.2 (~P9 in mice) marks a critical period for this. Further safety and efficacy data are necessary to move forward with clinical trials.

Another potential treatment, gapmer ASOs, have been utilized to alleviate seizures in *Scn2a* ^+/*R1883Q*^ mice.^56^ Using an effective dose (ED) that reduced *Scn2a* mRNA by 50% (ASO ED_50_) at P1 did not impair weight, sociability, or motor behavior in wild‐type mice and extended the life of *Scn2a* ^+/*R1883Q*^ mice by reducing seizures.^56^ However, ASO ED_50_ reduced anxiety‐like behaviors in *Scn2a* ^+/*R1883Q*^ mice beyond the effect seen in treated wild‐type animals,^56^ suggesting that there is an upper safety limit in ASO dosing for *Scn2a*. Treatment around P14 to P16 was still effective in *Scn2a* ^+/*R1883Q*^ mice, which suggests some flexibility in the treatment window.^56^ These data indicate that it is plausible for ASO therapy to treat some *SCN2A* variants effectively.

Currently, there is a gapmer ASO therapy being trialed in a pre‐term infant with early‐onset *SCN2A*‐related developmental and epileptic encephalopathy.^81^ The therapy, thus far, appears to be successful in reducing seizure occurrence in the infant.^81^ However, ASO therapies must be tailored to specific mutations, which may limit the possibility of generating a single, large‐scale treatment utilizing this approach.^82^ Furthermore, long‐term safety and efficacy studies are very important for SRD treatments, such as ASO therapy, as they have been and will likely continue to be administered to children.

### 5.2 Other treatments

Several pharmacotherapies have been tried in *Scn2a* models. Intraperitoneal injection of CX516, a positive allosteric modulator of α‐amino‐3‐hydroxy‐5‐methyl‐4‐isoxazolepropionic acid (AMPA) receptors, reduced hyperactivity of *Scn2a* ^*+/−*^ mice compared to untreated *Scn2a* ^*+/−*^ mice without adverse effects.^74^ Anti‐epileptic drugs, like ranolazine and GS967/PRAX‐562, which inhibit persistent sodium currents, reduce seizure frequency in F1.*Scn2a* ^*Q54*^ mice, but GS967/PRAX‐562 was more effective.^83^ GS967/PRAX‐562 improved survival of F1.*Scn2a* ^*Q54*^ mice, prevented hilar neuron loss, and protected the mice from induced seizures in the maximal electroshock paradigm^83^ but it also suppressed hippocampal mossy fiber sprouting, which could impair behaviors like spatial learning.^84^, ^85^ More data from GS967/PRAX‐562‐treated animals are necessary to elucidate adverse effects. These sodium channel blockers appear effective in treating seizures caused by *Scn2a* GOF mutations, but may be ineffective or harmful for *Scn2a* LOF variants.^18^, ^59^, ^86^, ^87^, ^88^

Deep brain stimulation (DBS) is another potential avenue of seizure treatment for at least some individuals with pathogenic *SCN2A* variants. Low‐frequency stimulation (3 Hz) of the ventral hippocampal commissure in *Scn2a* ^*Q54*^ mice reduced seizure severity, duration, and frequency.^89^ Subsequently, DBS has been applied successfully to a 3‐year‐old female *SCN2A* patient with medically refractory dystonia.^90^ The DBS electrodes were placed bilaterally in the globus pallidus interna, and a stimulation pattern of 2.0 mA, 125 Hz, and 60 μs pulse width was utilized.^90^ Six months post‐surgery, her dystonia appeared to be well controlled, with little reliance on rescue medications, and her overall well‐being seems to have drastically improved.^90^ These promising results support continued exploration of DBS for treatment of seizures and associated comorbidities in SRDs. Overall, it appears that early intervention has the greatest likelihood of success, although this may vary depending on the specific symptoms being targeted. For some symptoms, such as VOR gain and seizure attenuation, later intervention may be effective as well.

## 6 OTHER RODENT MODELS

Investigations in rats established our foundational understanding of *SCN2A*, like the temporal patterning of expression^91^ and the subcellular localization of Na_v_1.2.^92^ This work showed that Na_v_1.2 has a complementary expression pattern with Na_v_1.1 throughout the rat cortex and is preferentially localized to axons.^91^ Further work revealed a population of Na_v_1.2 lacking the beta‐2 subunit during the first 2 weeks of life that disappeared in older animals, indicating that *Scn2a* undergoes developmental stage‐specific changes.^91^, ^92^ Recently, a Long–Evans rat *Scn2a* ^*+/−*^ model was engineered using CRISPR‐Cas9 to generate a frameshift mutation disrupting exon 5.^93^ These *Scn2a* ^*+/−*^ rats display impairments in spatial alternation‐related behaviors.^93^

An *Scn2a* ^*+/−*^ prairie vole with premature stop codons in the first and third exons has been generated.^94^ Prairie voles have social structures more similar to humans than rats or mice and may better emulate the social changes seen in human SRDs. Preliminary data from *Scn2a* ^*+/−*^ prairie voles indicate impaired dendritic excitability similar to *Scn2a* ^*+/−*^ mice.^94^ This may be the first evidence that dendritic deficits caused by loss of *Scn2a* are conserved across species.

## 7 DISCUSSION

SRDs can drastically change a child's quality of life; therefore, it is imperative to determine the pathologies and prognoses of *SCN2A* variants. The LOF or GOF classification system simplifies the categorization of SRDs but fails to capture the nuances.^1^, ^16^, ^19^ There are *SCN2A* variants that display both LOF and GOF characteristics, which defies the binary, like p.K1422E.^22^ Furthermore, data from the existing non‐human mammalian models of SRDs, as well as clinical data, support the idea that phenotypes in SRDs are heavily influenced or driven by factors outside of a variant's channel kinetics.^39^, ^55^, ^65^, ^68^ Moreover, in the rare cases where identical pathogenic *SCN2A* variants have occurred in different people, the patients still have distinct symptom profiles^16^, ^21^, ^24^, ^67^—for instance, the p.L1650P variant presented in a male child as episodic ataxia but in the father as episodic hemiplegia.^67^ Similarly, the p.R1319Q and p.V261M variants cause severe developmental delay in some people but not others.^95^, ^96^ The p.R102X and p.R1435X variants cause seizures in some, but not all.^12^, ^21^, ^97^, ^98^ This heterogeneity in symptom presentation is also evident in the data from the behavioral and seizure studies across the existing non‐human mammalian models of SRDs, even for those with similar, if not identical, mutations. A LOF or GOF classification may guide initial treatment, but it is unlikely to capture every individual's complete biophysical and symptom profile. Furthermore, relying solely on the “LOF” or “GOF” label of an *SCN2A* channel variant can lead to misapplication of treatments and adverse effects.^18^, ^61^, ^88^ It is important to note that there is growing interest in the field in investigating mechanisms beyond channel kinetics that contribute to SRDs.^24^

When conducting behavioral, seizure, or electrophysiological investigations in non‐human mammalian models of SRDs, beyond basic channel kinetics, it is paramount to consider the degree of Na_v_1.2 loss/gain, the cell type, developmental stage/age, sex, and/or brain region being assessed, since the functional role of *Scn2a* changes across these conditions. For instance, evaluating seizure phenotypes across *Scn2a* mouse models reveals that seizures in SRDs have etiologies related to the developmental stage, cell type, and brain region.^33^, ^34^, ^50^, ^51^, ^55^, ^56^ Furthermore, multiple *SCN2A* variants have demonstrated a shifting biophysical profile depending on the Na_v_1.2 isoform present, and some can exhibit greater dysfunction while the neonatal isoform is expressed.^8^, ^60^, ^61^, ^98^ In addition, rodent studies that assessed multiple ages also found that phenotypes shift over time,^45^, ^74^, ^75^ and multiple studies have identified electrophysiological alterations that are present only during the first postnatal week.^31^, ^33^, ^34^ These data, along with others, indicate that the cell type and developmental stage of an animal alter how Na_v_1.2 dysfunction presents.^28^, ^31^, ^38^, ^44^ Thus, knowing and reporting the age and/or developmental stage of assessment, for a model or patient, is crucial for interpreting data. Of note, there are only two published studies that have investigated behavior (specifically, signs of early communication) during the early postnatal period.^45^, ^74^ Therefore, there is a paucity of information regarding how alterations in Na_v_1.2 shape phenotypes over the lifetime, indicating the need to study the developmental effects and trajectory of the Na_v_1.2 channel. To address this gap, *Scn2a* models should be explored from a developmental perspective with close and accurate reporting of the ages of animals evaluated. It is also important to remember that *SCN2A* is not a sex‐chromosome‐linked gene; therefore, SRDs are equally likely to occur in males and females. However, multiple studies of *Scn2a* rodent models only reported on one sex,^30^, ^38^, ^45^, ^52^, ^73^, ^74^, ^76^ but investigations that included both sexes often reported sex‐specific behavioral phenotypes.^22^, ^28^, ^57^ Investigations of sex differences in humans may not be feasible, as specific pathogenic *SCN2A* variants are often de novo and occur at low rates^10^, ^17^, ^24^; however, investigations utilizing non‐human mammalian models of SRDs can be powered to evaluate sex differences. Knowing potential differences in SRD symptom presentations can empower healthcare providers to better recognize and treat SRDs across all patients.

Currently, only four of the existing *Scn2a* rodent models emulate a human variant.^22^, ^34^, ^38^, ^50^ As *SCN2A* variants are often de novo, generating a model of every human variant is not feasible. However, models can be generated for variants that have the highest rate of occurrence, or that occur near known or putative post‐translational modification sites. Having more human‐relevant *Scn2a* rodent models would be an invaluable tool for further identification of disease modifiers to target for treatment. Regardless, the current non‐human mammalian models have provided foundational information about isoform differences and the cell‐ and brain region‐specific roles of Na_v_1.2, and have moved us closer to treatment options for these devastating conditions.

## AUTHOR CONTRIBUTIONS

K.E.J.S. provided the conceptualization for this manuscript. M.F.H.A. and K.E.J.S. created the figures and supplemental tables. K.E.J.S., M.F.H.A., and A.J.W. contributed to the literature review and wrote the manuscript. The final version was approved by all authors.

## FUNDING INFORMATION

This work was supported by Roy J. Carver Trust, Carver College of Medicine, and iDREAM (R25 NS130966) to M.F.H.A., the Neuroscience Graduate Program T32 (T32 NS007421) to K.E.J.S., and the Department of Defense Autism Research Program (DOD AR220030) to A.J.W.

## CONFLICT OF INTEREST STATEMENT

None of the authors has any conflict of interest to disclose.

## ETHICAL STATEMENT

We confirm that we have read the Journal's position on issues involved in ethical publication and affirm that this report is consistent with those guidelines.

## Supporting information


Table S1.

Table S2.

## Data Availability

Data sharing is not applicable to this article, as no new data were created or analyzed in this study.
